# Editorial: Venoms and Toxins: Functional Omics and Pharmacological Insights

**DOI:** 10.3389/fphar.2022.887513

**Published:** 2022-04-27

**Authors:** Choo Hock Tan, Kae Yi Tan, Timothy N. W. Jackson

**Affiliations:** ^1^ Venom Research and Toxicology Laboratory, Department of Pharmacology, Faculty of Medicine, University of Malaya, Kuala Lumpur, Malaysia; ^2^ Protein and Interactomics Laboratory, Department of Molecular Medicine, Faculty of Medicine, University of Malaya, Kuala Lumpur, Malaysia; ^3^ Australian Venom Research Unit, Department of Biochemistry and Pharmacology, University of Melbourne, Parkville, VIC, Australia

**Keywords:** venom proteome, Indian red scorpion, cobra, alpha-neurotoxin, Australian brown snake, phospholipase A2, habu snake, thrombin-like serine protease

Venoms are mixtures of toxins—bioactive molecules that serve predatory, digestive and defensive functions vital for the survival of venomous animals. This Research Topic contains two review articles and four original research papers, highlighting recent advancements in venomics and its applications in the functional characterization and medical importance of venoms and toxins.

In the review article by Das et al. (2021), the medical importance of the Indian red scorpion (*Mesobuthus tamulus*), a potentially deadly but rather understudied species from the Indian sub-continent, was described. The authors summarized the research progress made toward understanding the *M. tamulus* venom proteome over the years, and correlated the venom proteome with the pathophysiology of envenoming resulting from its sting. The poor immunorecognition and neutralization by scorpion antivenoms of the low molecular mass toxins in the venom were highlighted. In addition, the proteomic analysis unveiled the richness of pharmacologically active molecules in the species’ venom, suggesting its potential for drug discovery. The second in-depth review article, by Tasoulis et al. (2022), explored the current state of venom proteomic methods, associations between methodological and biological variability and the diversity and abundance of protein families, reported for snake venom proteomes from September 2017 to April 2021. The review included a total of 81 venom proteomic studies of 79 snake species, where relative toxin abundance was reported for 70 species and toxin diversity (number of different toxins) was reported for 37 species. The review underscores the need to clarify the differences in the data resultant from distinct methodological approaches and advocates for standardized protein classification, nomenclature and reporting procedures in the rapidly evolving research field of venomics.

The Research Topic also includes research papers on the venom proteomes of two lesser-known cobra species: *Naja samarensis* from the southern Philippines (Palasuberniam et al., 2021), and *Naja sagittifera* from Nicobar Island, India (Attarde et al., 2021). Short-chain alpha-neurotoxins (SαNTX) were the most highly expressed (66% of the total venom proteins) principal lethal component in *N. samarensis* venom, but were only weakly cross-neutralized by commercially available antivenom raised against venom from the Philippine Cobra (*Naja philippinensis*). Similar problems were reported for *N. sagittifera* venom, which contains more long neurotoxins but the venom is poorly cross-neutralized by existing Indian antivenoms, which are raised against the subcontinent’s “Big Four”. The phylogenetic study showed that *N. sagittifera* is more closely related to the monocled Cobra (*Naja kaouthia*) than it is to *N. naja* (its closest relative amongst the Big Four); however, the Thai Monocled Cobra Antivenom was also found to be poor in neutralizing its venom. These two studies of poorly known species of cobra thus come to similar conclusions. Both unravel the toxin compositions of the venoms and highlight the need for species-specific antivenom products to improve the treatment of snakebite envenoming in the Philippines and India.

The article by Vuong et al. (2021) provided insight into the toxic activities of venom proteins, specifically phospholipases A_2_ (PLA_2_), from Australian brown snakes (genus *Pseudonaja*), which are responsible for a majority of life-threatening bites in that country. The authors demonstrated that PLA_2_ toxins are implicated in *Pseudonaja aspidorhyncha*, *Pseudonaja nuchalis* and *Pseudonaja textilis* venom-induced vasorelaxation and the sympatholytic effects of *Pseudonaja affinis* and *Pseudonaja textilis* venoms. There were little to no direct venom-induced cardiac effects observed, hence it was proposed that cardiovascular collapse following brown snake envenoming is likely because of a fall in mean arterial pressure instead of a specific mechanism for inducing cardiac toxicity. In demonstrating the abrogation of the PLA_2_-mediated toxic effects of *Pseudonaja* venoms by the small molecule phospholipase A_2_ inhibitor Varespladib, their finding also supports the potential use of this drug as an initial (pre-referral) and/or adjunct (in combination with antivenom) therapeutic agent for brown snake envenoming. Further pre-clinical (e.g., *in vivo*), and ultimately clinical, research will be required to validate this possibility.

In southern Japan, envenoming by the habu snake (*Protobothrops flavoviridis*) causes severe tissue damage due to the presence of myonecrotic toxins in the venom. Ogawa et al. (2021) conducted a focused proteomics analysis of habu venom proteins, and identified a thrombin-like serine protease, TLSP2 (TLf2). This toxin, which is a proteolytic isoform that is inactive in isolation, functions as a myonecrosis-enhancing factor by interacting synergistically with Lys49-PLA_2_ myotoxins in the venom. The catalytically inactive TLSP2 (TLf2) was demonstrated to be a target protein of antiserum that prevents habu venom-induced necrosis caused by the Lys49-PLA_2_. The finding highlights the fact that venoms are complex systems in which individual components often work together to potentiate toxic effects, and suggests that such synergistic properties of different toxins in a venom should be considered in the design and development of treatment for snakebite envenoming. For example, a synergistic co-factor may be more immunogenic or possess more stable epitopes than the toxin it chaperones or potentiates, making it a rational target for the design of targeted therapeutics.

Together, this Research Topic provides insights into the integration of “-omics” technologies in venom profiling, characterization and application. The knowledge gained shall contribute to a better understanding of the pathophysiology of envenoming, the strength and weakness of treatments (e.g. conventional antivenoms or small molecule inhibitors), and the potential use of toxins in drug discovery.
